# A monoclonal antibody against kininogen reduces inflammation in the HLA-B27 transgenic rat

**DOI:** 10.1186/ar1728

**Published:** 2005-04-04

**Authors:** James C Keith, Irma M Sainz, Irma Isordia-Salas, Robin A Pixley, Yelena Leathurby, Leo M Albert, Robert W Colman

**Affiliations:** 1 Department of Cardiovascular and Metabolic Diseases Research, Wyeth Research, Cambridge, Massachusetts, USA; 2 The Sol Sherry Thrombosis Research Center, Temple University School of Medicine, Philadelphia, Pennsylania, USA

## Abstract

The human leukocyte antigen B27 (HLA-B27) transgenic rat is a model of human inflammatory bowel disease, rheumatoid arthritis and psoriasis. Studies of chronic inflammation in other rat models have demonstrated activation of the kallikrein–kinin system as well as modulation by a plasma kallikrein inhibitor initiated before the onset of clinicopathologic changes or a deficiency in high-molecular-mass kininogen. Here we study the effects of monoclonal antibody C11C1, an antibody against high-molecular-mass kininogen that inhibits the binding of high-molecular-mass kininogen to leukocytes and endothelial cells in the HLA-B27 rat, which was administered after the onset of the inflammatory changes. Thrice-weekly intraperitoneal injections of monoclonal antibody C11C1 or isotype IgG_1 _were given to male 23-week-old rats for 16 days. Stool character as a measure of intestinal inflammation, and the rear limbs for clinical signs of arthritis (tarsal joint swelling and erythema) were scored daily. The animals were killed and the histology sections were assigned a numerical score for colonic inflammation, synovitis, and cartilage damage. Administration of monoclonal C11C1 rapidly decreased the clinical scores of pre-existing inflammatory bowel disease (*P *< 0.005) and arthritis (*P *< 0.001). Histological analyses confirmed significant reductions in colonic lesions (*P *= 0.004) and synovitis (*P *= 0.009). Decreased concentrations of plasma prekallikrein and high-molecular-mass kininogen were found, providing evidence of activation of the kallikrein–kinin system. The levels of these biomarkers were reversed by monoclonal antibody C11C1, which may have therapeutic potential in human inflammatory bowel disease and arthritis.

## Introduction

Human leukocyte antigen B27 (HLA-B27) transgenic Fisher rats are normal at birth but develop chronic inflammation of multiple organ systems as they age. Transgenic rats of this strain, overexpressing the human HLA-B27 and β_2_-microglobulin proteins, develop lesions of the gastrointestinal system, the joints, the skin, and the gonads, which seem similar to the spondyloarthropathies in humans that have been associated with the HLA-B27 and β_2_-microglobulin genes [1,2]. The gastrointestinal inflammation is mostly limited to the mucosa and submucosa, exhibiting histological features similar to those present in inflammatory bowel disease (IBD) [1-4]. Chronic intestinal inflammation is the first to occur, with clinical signs of diarrhea apparent after 12 weeks of age. About 4 weeks later, joint inflammation is seen, and these rats can also be used for a model of inflammatory arthritis [3].

**Figure 1:**
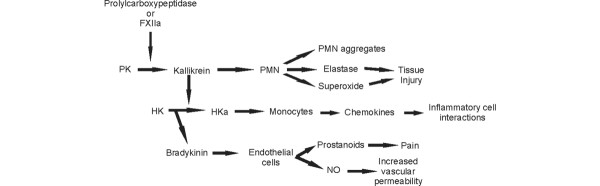
Kallikrein–kinin system (KKS). The KKS is initiated by factor XIIa (FXIIa) or prolylcarboxypeptidase on the endothelial cell and leukocyte (polymorphonuclear cell (PMN)) surface, generating the enzyme kallikrein, which in turn cleaves high-molecular-mass kininogen (HK) to yield bradykinin (BK) and cleaved high-molecular-mass kininogen (HKa). Kallikrein is chemotactic, aggregates neutrophils, and stimulates the release of elastase and superoxide (potent inducers of tissue injury). BK stimulates vasodilation, mediates pain through the release of prostaglandins, and stimulates vascular permeability through the generation of nitrous oxide (NO). PK, prekallikrein.

The plasma kallikrein–kinin system (KKS), which is initiated by factor XIIa [5] or prolylcarboxypeptidase [6] after binding of high-molecular-mass kininogen (HK) and plasma prekallikrein (PK) to the surface of endothelial cells and leukocytes [7], generates the enzyme kallikrein. Kallikrein in turn cleaves HK to yield the inflammatory mediators bradykinin (BK) and cleaved high-molecular-mass kininogen (HKa) [8]. Kallikrein is chemotactic, aggregates neutrophils [9], stimulates superoxide formation, and releases elastase from neutrophils [10], all of which induce tissue injury. BK stimulates vascular permeability and angiogenesis after binding to endothelial cells [11] and also mediates pain through the release of prostanoids [12]. HKa stimulates cytokine release from rat [13] and human monocytes[14]. Thus, activation of the KKS is an inflammatory stimulus that might be operative in human disease, as represented in Fig. 1.

We have shown that KKS activation mediates the acute and chronic phases of T cell-mediated arthritis induced by peptidoglycan–polysaccharide complexes from Group A streptococci (PG-APS) in Lewis rats [15] and is selectively activated in granulomatous enterocolitis in these susceptible rats, but not in resistant Buffalo rats [16]. We have discovered a genetic difference in kininogen structure between resistant Buffalo and Fischer F344 inbred rats and the susceptible Lewis rat that results in accelerated cleavage of HK in the latter. This mutation consists of a single nucleotide polymorphism coding for the amino acid alteration, S511N, in the HK gene of Lewis (N511) (mutant) versus Buffalo and Fischer (S511) (wild-type) rats that results in an altered glycosylation state [17] and an increased rate of HK cleavage by plasma kallikrein with release of BK. We have shown that BK has a critical role in the PG-APS-mediated arthritis [18]. We have also implicated BK receptors as having a role in a different model of IBD, indomethacin-induced colitis [19]. Most recently, we have shown that a monoclonal antibody (mAb), C11C1, acting to prevent HK interaction with cells involved in inflammatory disorders, inhibited the development of acute and chronic arthritis in the PG-APS model [20].

To demonstrate that this effect was not specific for a single model and to allow us to assess the possibility of treating established chronic inflammation, we examined an HLA-B27 transgenic rat model of chronic inflammation of the intestine and peripheral joints. Administration of mAb C11C1 ameliorated colitis and tarsal joint inflammation.

## Materials and methods

HLA-B27 transgenic male rats were purchased from Taconic Laboratories (Germantown, NY) and housed one per cage in accordance with Wyeth Research facility standard operating procedures. They received a standard regimen of food and water. Animals were thoroughly acclimated to the laboratory before the beginning of the study. The study was approved by the Wyeth Research (Cambridge) Institutional Animal Care and Use Committee.

At 23 weeks of age, 10 male rats presenting the clinical signs of colitis (diarrhea) and arthritis (erythematous and swollen hind paws) were randomized into either an isotype control mAb IgG (*n *= 5) or mAb C11C1 (*n *= 5) treatment group. Each rat was weighed daily and received an intraperitoneal injection of isotype IgG_1 _(6 mg/kg) or mAb C11C1 (1.9 mg/kg) three times per week for 16 days. Stool character observations for each animal on each day of study were assigned numerical scores of 3 for diarrhea, 2 for soft stool and 1 for normal stool. The clinical signs of arthritis in the tarsal joints were monitored daily in all of the animals. This assessment was performed visually with a scale for swelling (0 to 3) and for erythema (0 to 3) of the hindpaws (normal paw = 0, mild = 1, moderate = 2, severe = 3). The maximum possible score for arthritis per animal per paw per day was 6 (total per animal = 12 for both hindpaws).

### Histological analyses

At the end of the experiment, the animals were killed with 100% carbon dioxide, and the distal 10 cm of colon of each rat was removed and opened. Four standardized samples of colon were immersed in 10% neutral buffered formalin [21]. Samples from each rat were prepared for histological evaluation. The formalin-fixed tissues were processed in a Tissue Tek vacuum infiltration processor, Model 4617 (Miles, Inc., West Haven, CT) for paraffin embedding. The samples were sectioned at 5 μm thickness and then stained with hematoxylin and eosin (H&E) for histological evaluation. Histological lesions were assigned scores in accordance with a previously defined scoring scheme [21-24]. In brief, the severity in the colonic sections was evaluated for ulcer size (none = 0, small = 1, large = 2), degree of inflammation (none = 0, mild = 1, moderate = 2, severe = 3), depth of lesion (none = 0, submucosa = 1, muscularis propria = 2, involving serosa = 3), and fibrosis (none = 0, mild = 1, severe = 2). The total histological scores for the colon specimens ranged from 0 to 10.

During necropsy, segments of the rear limbs (with the tarsal joints) were removed, fixed in 10% buffered formalin, and examined as described previously [22]. After decalcification, histological sections were obtained and stained with H&E or Safranin O/Fast Green stain. Synovial tissue from tarsal joints was evaluated on the basis of synovial hyperplasia (synovial cell proliferation: mild = 1, moderate = 2, villus formation = 3), fibroplasia (subsynovial fibrosis: minimal = 1, one-third to one-half of areolar tissue replacement = 2, whole thickness areolar tissue replacement = 3), inflammatory cell infiltrates (occasional = 0, small numbers/around blood vessels = 1, small focal collections = 2, large foci = 3), and pannus formation (organizing inflammatory exudates within the joint space: nondetectable = 0, detectable = 2). The total histological score for synovial inflammation ranged from 0 to 11 [25]. Articular cartilage was evaluated with Mankin's histological grading system [26]: cartilage organization changes (normal = 0, surface irregularity = 1, pannus and surface irregularity = 2, clefts to transitional zone = 3, clefts to radial zone = 4, clefts to calcified zone = 5, complete disorganization = 6), chondrocyte proliferation (none = 0, hypercellularity = 1, cloning = 2, hypocellularity = 3), proteoglycan contents (Safranin O/Fast Green staining, normal = 0, slight reduction = 1, modest reduction = 2, severe reduction = 3, no dye noted = 4), and tidemark integrity (intact = 0, crossed by blood vessels = 1). The total Mankin score ranged from 0 to 14. Histological H&E-stained sections taken from kidney, liver, and spleen from the mAb C11C1-treated group were evaluated for signs of systemic inflammation and/or toxicity.

### Blood collection

Blood samples were obtained by cardiac puncture with a 19-gauge, 3/4-inch needle on a 10 ml polypropylene syringe (BD Medical Systems, Franklin Lakes, NJ). The sample was obtained from the left atrium as the heart beat. The sample of 3 to 5 ml was obtained by slow vacuum (to prevent hemolysis) within a minute (to prevent clotting in the syringe). The blood was then transferred into pre-marked, 1 ml Eppendorf polypropylene tubes (Fisher Scientific, Pittsburgh, PA) containing 100 μl of anticoagulant (citrate-phosphate-dextrose solution with adenine, Sigma C-4431; Sigma Chemical Co.) to a final volume of 1 ml and gently mixed. Plasma was isolated by double centrifugation of the citrated blood in polypropylene tubes (Fisher Scientific) at 23°C. Aliquots were stored at -70°C until assayed.

### Assays of KKS activation *ex vivo*

PK function levels were performed by a microtiter, amidolytic assay using a chromogenic substrate, S-2302 (Pro-Phe-Arg-*p*-nitroanilide; Chromogenix, Moindal, Sweden), as described previously [27]. HK coagulant activity was evaluated by our modification of an APTT test assay [28,29], using total kininogen-deficient plasma purchased from George King (Overland Park, KS) [19]. In addition, factor XI and factor XII coagulant activity assays were performed with a similar method using the appropriate deficient plasma obtained from George King.

### Statistical analyses

All the evaluations were made by examiners blinded to the treatment groups. All of the parameters were subjected to Students' t test between groups. Data were expressed as means ± SEM, and differences were deemed significant if *P *< 0.05.

## Results

**Figure 2:**
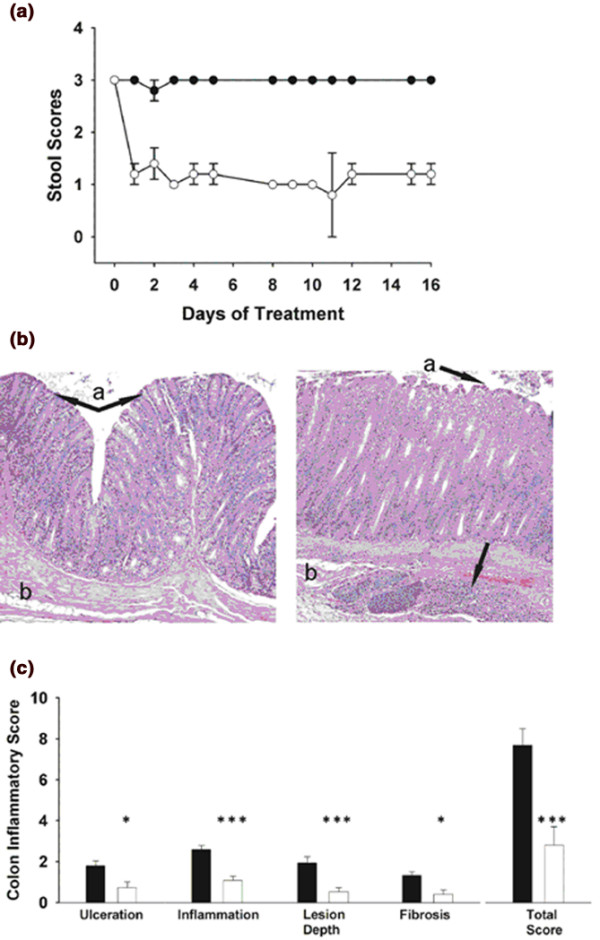
Effect of mAb C11C1 on HLA-B27 transgenic rats colonic inflammation. **(a) **Effects of monoclonal antibody (mAb) C11C1 on diarrhea in human leukocyte antigen B27 (HLA-B27) rats. Stool score was determined five times a week (normal stool = 1, soft stool = 2, watery stool = 3). mAb C11C1 (1.9 mg/kg) was administered three times a week for 16 days. The control group received murine isotype IgG_1 _(6 mg/kg) three times a week for 16 days. All stool scores are significantly different between the two groups for each corresponding day (*P *< 0.005) except for day 11 (*P *= 0.03). Data are shown as means ± SEM. Filled circles, IgG_1_-treated group; open circles, mAb C11C1-treated group. **(b) **Effects of mAb C11C1 on colonic mucosa in HLA-B27 rats. Photomicrographs of representative sections of colon from C11C1-treated (left) and IgG-treated (right) HLA-B27 transgenic rats. Note the extensive inflammatory cell infiltrates within the mucosa (a) and submucosa (b) with loss of villus formation on the mucosal surface indicated by the arrow (a) in the IgG group (right) compared with the C11C1 group (left). The branched arrow (left) points to the villus formation normally present in the colon (mAb C11C1-treated group). Hematoxylin and eosin stain; original magnification × 100. **(c) **Effects of mAb C11C1 on colonic inflammatory changes in HLA-B27 rats. mAb C11C1 decreased inflammatory changes in the colonic sections as evaluated by ulceration (*P *= 0.02), inflammation (*P *< 0.001), depth of lesion (*P *= 0.004), and degree of fibrosis replacement (*P *= 0.01) compared with IgG_1 _administration. Treatment with mAb C11C1 (open bars) significantly decreased the extent and intensity of the total colonic inflammatory score (*P *= 0.004). Data are shown as means ± SEM. *P < 0.05; ***P < 0.005.

Twenty-four hours after the onset of therapy in the mAb C11C1-treated rats, the clinical signs of intestinal inflammation (diarrhea) had disappeared, and the stool character remained normal or nearly normal for the duration of the experiment (Fig. 2a). Histological analysis demonstrated significant reductions (from 60 to 75%) in lesion scores in the animals treated with mAb C11C1 in comparison with animals injected with isotype IgG_1 _(Fig. 2b,c).

**Figure 3:**
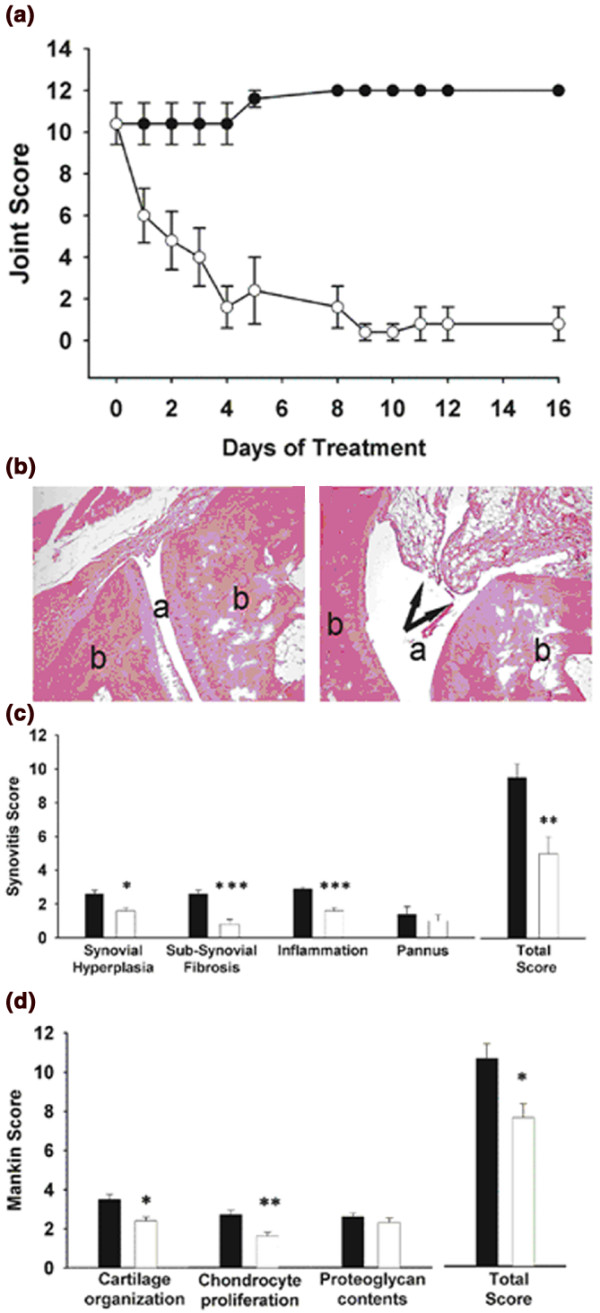
Effect of mAb C11C1 on HLA-B27 transgenic rat inflammatory arthritis. **(a) **Effects of monoclonal antibody (mAb) C11C1 on clinical signs of arthritis in human leukocyte antigen B27 (HLA-B27) rats. mAb C11C1 was administered at the same dose and frequency as in Fig. 2a. Mean joint score was determined daily, except at weekends. All joint scores are significantly different between the two groups for each corresponding day (*P *< 0.001) except for days 1 (*P *> 0.03), 2 (*P *= 0.01) and 3 (*P *= 0.006). Data are shown as means ± SEM. Filled circles, IgG1-treated group; open circles, mAb C11C1-treated group. **(b) **Effects of mAb C11C1 on joint histology in HLA-B27 rats. Photomicrographs of representative sections of tarsal joints from C11C1-treated (left) and IgG-treated (right) HLA-B27 transgenic rats. Note the clear joint space (a) and normal appearance of bone (b) in the mAb C11C1-treated group (left) compared with the inflamed villus formation (arrows) occupying the synovial space (a) in the IgG-treated group (right). Hematoxylin and eosin stain; original magnification × 100. **(c) **Effects of mAb C11C1 on synovial inflammatory changes in HLA-B27 rats. Treatment with mAb C11C1 (open bars) decreased synovial proliferation (hyperplasia) (*P *= 0.01), subsynovial fibrosis (fibroplasia) (*P *= 0.001), and degree of inflammation (*P *< 0.001), but not pannus formation. The total score of the control IgG1 of 9.6 ± 1.0 was reduced by mAb C11C1 to an inflammatory score of 5.0 ± 1.0 (*P *= 0.009). Data are shown as means ± SEM. *P < 0.05; **P < 0.01; ***P < 0.005. **(d) **Effects of mAb C11C1 on cartilage and bone inflammatory changes in HLA-B27 rats. mAb C11C1 (open bars) significantly improved (decreased the Mankin score of) the cartilage organization (*P *= 0.01) and the altered chondrocyte proliferation (*P *= 0.008). The proteoglycan cartilage contents (Safranin O/Fast Green staining) were similar in both experimental groups (*P *> 0.05) and the tidemark integrity was preserved (data not shown). The total Mankin score was significantly decreased in the mAb C11C1-treated group (*P *= 0.02). Data are shown as means ± SEM. *P < 0.05; **P < 0.01; ***P < 0.005.

Daily visual inspection of the tarsal joints in the mAb C11C1-treated animals revealed marked reductions in the degree of swelling and erythema of the joints compared with isotype-treated animals. As can be seen in Fig. 3a, within 24 hours of the onset of therapy, the mean joint histological scores in the mAb C11C1-treated rats decreased by about 50% compared with the mAb control group. By the end of 1 week of treatment, the clinical signs of arthritis had almost disappeared. Evaluation of the histological features of the arthritis in the tarsal joints at the termination of the experiment on day 16 showed a marked reduction in the parameters of synovitis in the rats treated with mAb C11C1 compared with those receiving isotype IgG_1 _(*P *< 0.05) (Fig. 3b,c). In a similar manner to the changes seen in the colon, 40 to 60% decreases in the various components of the synovitis score occurred. However, the effects on the articular cartilage were more modest. Nevertheless, the cartilage organization, chondrocyte proliferation and total Mankin score were significantly decreased (Fig. 3d). Tidemark integrity was preserved in all groups (data not shown). Histological analysis of kidney, liver and spleen sections showed normal architecture without any signs of inflammation or toxicity in both treated groups (results not shown).

### KKS activation assays

**Figure 4:**
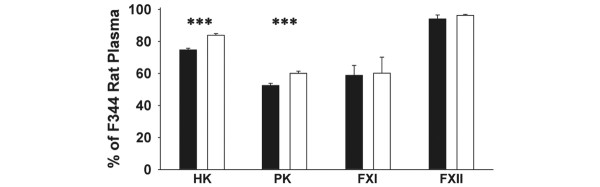
Kallikrein–kinin system (KKS) assays. Plasma KKS protein concentrations in the human leukocyte antigen B27 transgenic rats treated with control monoclonal antibody IgG (filled bars) or monoclonal antibody C11C1 (open bars) at day 16 of the experimental protocol. Values were compared with a pool of normal Fischer 344 rat plasma. Both high-molecular-mass kininogen (HK) and prekallikrein (PK) were significantly decreased in the IgG1-treated group and were closer to normal in the C11C1-treated group. Both experimental groups showed decreased factor XI (FXI) with no significant differences between them. There were no significant changes between any groups in factor XII (FXII). ***P < 0.005.

To assess KKS system activation in this animal model of inflammation, we compared the experimental groups' results with a standard pool of normal Fischer 344 rat plasma (Fig. 4). We measured the plasma functional levels of four contact proteins. At the termination of the experiment (day 16), HK levels were reduced in both groups compared with the standard pool level. The values in the mAb C11C1-treated animals were closer to normal than those in the isotype-treated animals. HK levels were significantly lower in the isotype IgG-treated group (74.7 ± 1.0) than in the group receiving mAb C11C1 (83.9 ± 1.1) (*P *< 0.001). PK levels were significantly decreased in the IgG isotype group (52.5 ± 1.3%) versus the mAb C11C1-treated group (60.1 ± 1.3%; *P *< 0.005). Factor XI was similarly lower in both experimental groups but factor XII was not lower (in any group). Neither difference in factor XI or factor XII levels between the two experimental groups was significant. The results of these assays were similar to those observed in our previous studies [20], in which a decrease in HK and PK was the most consistent evidence for KKS activation.

## Discussion

Therapy with C11C1, a mAb that interferes with the cellular binding of HK, evoked marked anti-inflammatory activity in both the colon and the tarsal joints of HLA-B27 transgenic rats. The onset of anti-inflammatory activity by mAb C11C1 was rapid and sustained throughout the study, with the first effect seen in the intestine. The joint changes began to resolve with improvement in stool character, but it took almost 10 days for the joint swelling and erythema to reach minimal levels (as reflected in joint score values). The histological effects in the colon seemed to be more complete than those seen in the tarsal joints because only a modest effect was seen on the articular cartilage lesions, as reflected in the Mankin score. However, if one compares the colonic score results with the synovitis score results, the effect was very similar in both character and magnitude. The isotype IgG_1 _group KKS assays showed a decrease in HK and PK levels consistent with this system activation, whereas the mAb C11C1-treated group showed significantly increased levels of both proteins. These observations are explained by the fact that mAb C11C1 inhibits the activation of HK, thus blocking KKS activation and decreasing the signs of inflammation [20].

The HLA-B27 transgenic rat model has been used for several years to evaluate the activity and mechanisms of actions of anti-inflammatory molecules [22,23,30-34]. This model is very reproducible and consistent, as long as the environmental conditions remain stable. The chronic inflammation seen in these transgenic rats seems to be the result of HLA-B27 transgene expression-induced alterations in antigen processing and subsequent immune responses to the microbial environment in the lumen of the animal's gastrointestinal tract [35,36]. These aberrant responses lead to CD4^+ ^T cell activation and proinflammatory cytokine production. Broad-spectrum antibiotic therapy can produce significant remissions of the inflammatory lesions, but relapse occurs when antibiotic therapy stops [35]. If antibiotic therapy is followed by inoculation of the gut with probiotic agents such as *Lactobacillus rhamnosus*, relapse is prevented [36]. Lactobacilli have also been shown to be effective in treating patients with chronic pouchitis after ileal pouch–anal anastomosis for the treatment of ulcerative colitis [37].

In addition to antibiotics and probiotic agents, other standard anti-inflammatory agents used in the long-term treatment of IBD patients are also active in the HLA-B27 transgenic rat. Both dexamethasone and prednisolone produce dose-dependent reductions in the inflammation in these animals [38,39]. As in patients with IBD, sulfasalazine at low doses is without effect in the HLA-B27 transgenic rat [40], but high doses do ameliorate the disease [41].

Three approaches have been used in our laboratory to show that the KKS has a major role in inflammatory arthritis and enterocolitis with the use of the PG-APS models. First, we used a specific oral reversible tight-binding active-site inhibitor of plasma kallikrein, D-Pro-Phe-boro-Arg. This specific kallikrein inhibitor attenuated acute inflammatory changes (edema, and neutrophil infiltration) and prevented arthritis and chronic systemic complications (splenomegaly, hepatomegaly, leukocytosis and the acute-phase reaction) in the PG-APS model [42]. The same plasma kallikrein inhibition modulated acute intestinal changes [28] as well as chronic granulomatous intestinal inflammation [29] similar to human Crohn's disease. Second, we showed that antagonists of BK receptor type 2 ameloriate acute arthritis [43] whereas an antagonist of BK receptor type 1 aggravated the joint inflammation [44]. We have recently shown that BK receptor antagonists can upregulate or downregulate specific cell-adhesion molecules [44]. Third, kininogen deficiency was first described in Brown Norway rats [45]. We introduced this mutation into a Lewis genetic background with five generations of backcrosses and showed that the deficiency of kininogen ameliorated acute and chronic enterocolitis [46]. Because we have previously successfully used the mAb C11C1 to inhibit tumor growth in a syngeneic murine model (Sainz IM, Isordia-Salas I, Pixley RA, Colman RW, unpublished work) and in a human colon carcinoma grown in a nude (immunodeficient) mouse model [47], we used this fourth approach in the present study. This antibody has recently been successfully employed in the PG-APS model in which mAb C11C1 inhibited inflammatory changes in joints, systemic inflammation, and activation of the kallikrein–kinin system [20]. Here we have demonstrated its efficiency in treating HLA-B27-associated inflammatory disease.

Each of the previous approaches to inhibiting the KKS to control inflammation was successful but had certain limitations. The plasma kallikrein active-site inhibitor displayed hepatic toxicity. The BK receptor antagonist had only a modest effect. Kininogen deficiency is rare in humans and is not really an applicable therapeutic modality. However, we were encouraged by the success of mAb C11C1 in the PG-APS model in the prevention of systemic and joint inflammation [20] and the lack of obvious side effects. The fact that antibodies against other inflammatory agonists have been used in the treatment of human IBD, arthritis and cancer make its use attractive. Until this study, mAb C11C1 had been used in a preventive mode. The HLA-B27 transgenic rat model permitted the rapid treatment of an established disease model. On the basis of these results, we suggest that mAb C11C1 might be a candidate for a therapeutic agent in human inflammatory disease.

## Conclusion

We have assessed a transgenic rat model in which the human gene encoding HLA-B27 has been overexpressed. These rats developed T cell-mediated, spontaneous arthritis resembling reactive or inflammatory arthritis. We were able to successfully treat an established disease with an antibody against kininogen without inducing side effects or toxicity in either the rat or the mouse model of the disease.

## Abbreviations

BK = bradykinin; H&E = hematoxylin and eosin; HK = high-molecular-mass kininogen; HKa = cleaved high-molecular-mass kininogen; HLA-B27 = human leukocyte antigen B27; IBD = inflammatory bowel disease; KKS = kallikrein–kinin system; mAb = monoclonal antibody; PG-APS = peptidoglycan–polysaccharide polymers from group A streptococci; PK = prekallikrein.

## Competing interests

The author(s) declare that they have no competing interests.

## Authors' contributions

JCK planned and supervised the entire animal protocol. He also participated in the statistical analysis and writing of the clinical results section and in the editing of the manuscript. IMS assessed the potential toxic effects of the treatment on kidney, lungs and liver. She also prepared the final version of all figures and collaborated in the statistical analysis, editing, and typing of the manuscript. IIS performed the KKS assays and, together with RAP, purified the antibody. RAP also participated in the statistical analysis, editing of the manuscript, and preparation of the KKS figure. YL performed the animal protocol and collected the data. LMA participated in the planning and execution of the animal project. RWC planned and initiated the entire product, wrote the introduction and discussion portions of the manuscript, and was responsible for final editing. All authors read and approved the final manuscript.
